# Nomogram for predicting survival after lymphatic metastasis in esophageal cancer: A SEER analysis

**DOI:** 10.1097/MD.0000000000034189

**Published:** 2023-08-04

**Authors:** Shuo Sun, Wenwen Yang, Yanjiang Yang, Mengmeng Fan, Feng Wang, Li He, Biao Han, Chang Chen

**Affiliations:** a The First Clinical Medical College, Lanzhou University, Lanzhou, Gansu Province, China; b Department of Thoracic Surgery, the First Hospital of Lanzhou University, Lanzhou, Gansu Province, China; c Qilu hospital of Shandong University, Shandong University, Jinan, Shandong province, China; d School of Management, Lanzhou University, Lanzhou, Gansu Province, China; e Gansu Province International Cooperation Base for Research and Application of Key Technology of Thoracic Surgery, The First Hospital of Lanzhou University, Lanzhou, Gansu Province, China.

**Keywords:** cox regression, esophageal cancer, nomogram, prognosis, SEER program

## Abstract

Lymphatic metastasis (LM) is a significant mechanism for the spread of esophageal cancer (EC) and predicts the poor prognosis of EC patients. This research aimed to assess the survival of patients with LM from EC by developing a nomogram. In this retrospective study, EC patients with LM from 2004 to 2015 in the Surveillance, Epidemiology, and End Results (SEER) database were divided by year of diagnosis into a training cohort and a validation cohort. Univariate and multivariate Cox regression analyses were employed to determine the prognostic factors of LM, and a nomogram was constructed. The discrimination and calibration of the nomogram were compared by the C-index, area under the curve value, and calibration plots. The survival time difference was compared using Kaplan–Meier curves. A total of 11,695 patients with EC were included in this analysis. LM occurred in 56.5% (n = 6614) of EC patients. In the post-propensity score matching (PSM) cohort, patients with LM had significantly lower median overall survival (OS) than those without LM. Multivariate Cox regression was used to identify the eleven independent prognostic factors. The C-index was 0.709 in both the training and test sets, revealing the good predictive performance of the nomogram. Based on the results of calibration plots and the receiver operating characteristic (ROC) curve, we demonstrate the great performance of the prognostic model. The survival time of EC patients with LM was remarkably lower than that of EC patients without LM. The nomogram model established in this study can precisely predict the survival of EC patients with LM.

## 1. Introduction

As the most common thoracic malignancy after lung cancer, esophageal cancer (EC) has very high morbidity and mortality. According to Global Cancer Statistics 2020, EC accounted for 3.1% (n = 604,100) of new cases and 5.5% (n = 544,076) of new deaths.^[1]^ As 2 pathological types of EC, adenocarcinoma (AC) and squamous cell carcinoma (SCC) have an uneven global distribution. SCC incidence in Southeast and Central Asia accounts for 79% of the global SCC incidence, while AC in Oceania, North America, Northern Europe, and Western Europe accounts for 46% of the global EAC incidence.^[2]^ Different subtypes of EC have different incidences in different parts of the esophagus. AC is more common in the lower esophagus, while SCC is more common in the upper esophagus.^[3]^ The incidence of esophageal AC has been on the rise in many Western countries in recent years.^[4,5]^ EC spreads rapidly once it develops. Lymphatic metastasis (LM) is one of the most common methods of EC metastasis, and its LM areas include mediastinal lymph nodes, cervical lymph nodes, and abdominal lymph nodes. The esophagus has a unique network of capillary lymphatic vessels, making the lymphatic drainage system extremely complex.^[6]^ Because the lymphatic vessels of the esophagus are located in the submucosa, LM is common in patients with EC, and approximately 52% of patients with EC have LM.^[7]^ Kenshi Kuge et al^[8]^ showed that there is a direct esophageal drainage system in the thoracic duct. Lymph spread plays an important role in the prognosis of EC patients.^[9]^ Lymph node status can effectively predict 5-year survival and recurrence of EC.^[10]^ Endoscopic ultrasonography, compute tomography, and positron emission tomography are common methods for diagnosing LM in EC.^[9]^ EC has a higher incidence of LM than other gastrointestinal cancers.^[11,12]^ Wen-Hu Hsu et al^[13]^ found that the number and proportion of lymphatic metastases are independent prognostic elements for EC patients. However, there is no nomogram model for forecasting the survival time of EC patients with LM. Therefore, we constructed a prognostic nomogram model to forecast 1-year, 3-year, and 5-year survival after LM in EC using data from the Surveillance, Epidemiology, and End Results Database (SEER) database between 2004 and 2015.

This study attempts to explore the factors influencing the prognosis of patients with EC and thus guide clinicians’ prognostic decisions and investigate the relationship between lymph node metastasis and prognosis in patients with EC. Establishing such a prognostic model may lead to earlier clinical interventions and benefits for EC patients, and the visualization of categories in the nomogram may help clinicians make more accurate judgments and treatments to improve postoperative survival time.

## 2. Methods

### 2.1. Patient selection

We extracted 11,695 patients with EC discovered between 2004 and 2015 from the SEER database, the largest cancer database in the United States. Included patients with EC must meet all following criteria: first malignant primary indicator and patients aged 19 to 85 with tumor size <600 mm. The exclusion criteria were as follows: incomplete follow-up date, incomplete clinicopathological information, and patients diagnosed by autopsy. Finally, this study included 11,695 patients diagnosed with EC, of whom 6614 developed lymphatic metastases. We included patients with EC from 2005 to 2007, 2009 to 2011, and 2013 to 2015 in the training cohort (n = 5039) and patients diagnosed in 2004, 2008, and 2012 in the validation cohort (n = 1575). We extracted race, N stage, primary tumor site, sex, grade, radiotherapy, histological type, T stage, chemotherapy, distant metastasis information, surgery, tumor size, age, and follow-up information from the SEER database. We assessed patient survival using overall survival (OS), which is the process from diagnosis to loss to follow-up or all-cause death. The best cutoff values for continuous variables, including tumor size and age, were determined by X-tile v3.6.1 (Yale University).^[14]^ This study applies AJCC TNM 6th edition staging. The SEER database is an open, deidentified database, so we do not require institutional review board approval.

### 2.2. Statistical analysis

We transformed continuous variables into categorical variables using X-tile software and compared differences in categorical variables using the chi-square or Fisher exact test. To exclude the effect of other variables on the prognosis of EC patients with or without LM, 1:1 propensity score matching (PSM) was performed in SPSS v26.0 (SPSS Inc.). Finally, 3697 patients with LM were matched with 3697 patients without LM. In the training cohort, we included factors with *P* < .05 in multivariate Cox regression from univariate Cox regression to identify independent prognostic factors. According to the results of multivariate Cox regression, a nomogram was established for predicting the prognosis of EC patients with LM. We also verified its validity by receiver operating characteristic (ROC) curve, C-index, calibration plots, and Kaplan–Meier curve analyses. All statistical analyses were performed in R software v4.1.3 (https://www.r-project.org/), GraphPad Prism v8.0.2 (GraphPad Software, Inc.), and SPSS v26.0 (SPSS Inc.). *P* values < .05 were considered statistically significant.

## 3. Results

### 3.1. Clinical features

We included 11,695 patients with EC diagnosed between 2004 and 2015 in this retrospective study. Their median follow-up was 15 months (interquartile range, 6–49 months). Demographic and all characteristics before and after PSM are shown in Table 1. In the pre- and post-PSM sets, the age of most patients is 23 and 67 years (60.4%, n = 7075; 59.9%, n = 4436), most patients had tumor size between 26 and 69 mm (55.2%, n = 6458; 59.0%, n = 4368), most patients were married (60.9%, n = 7127; 59.1%, n = 4373), and most patients received chemotherapy (70.0%, n = 8188; 73.7%, n = 5455), most patients received radiotherapy (64.2%, n = 7519; 68.5%, n = 5069), most patients did not receive surgery (56.2%, n = 6583; 59.1%, n = 4375), most patients did not develop distant metastasis (73.0%, n = 8538; 73.3%, n = 5420), and the histological type of most patients was AC (59.2%, n = 6931; 56.7%, n = 4195), The lower third is the most common site of EC (65.4%, n = 7653; 64.5%, n = 4772), and the majority of patients were white (84.4%, n = 9871; 83.3%, n = 6162) and male (81.2%, n = 9503; 79.7%, n = 5899), the most common T stage is stage T3 (42.9%, n = 5022; 43.1%, n = 3194), and the most common grade is Poorly differentiated (49.0%, n = 5739; 48.1%, n = 3562).

**Table 1:** Demographic characteristics before and after PSM.

		The pre-PSM cohort					the post-PSM cohort				
		Lymphatic metastasis		Non-Lymphatic metastasis		*P*	Lymphatic metastasis		Non-Lymphatic metastasis		*P*
		N	%	N	%		N	%	N	%	
		6614		5081			3697		3697		
Age	23–67	4216	63.74	2859	56.27	<.001	2270	61.40	2166	58.59	.036
	68–74	1313	19.85	1056	20.78		714	19.31	745	20.15	
	75–85	1085	16.40	1166	22.95		713	19.29	786	21.26	
Race	White	5606	84.76	4265	83.94	<.001	3088	83.53	3074	83.15	.048
	Black	573	8.66	547	10.77		358	9.68	408	11.04	
	Other	435	6.58	269	5.29		251	6.79	215	5.82	
Gender	Female	1118	16.90	1074	21.14	<.001	738	19.96	757	20.48	.582
	Male	5496	83.10	4007	78.86		2959	80.04	2940	79.52	
Primary Site	Upper third	295	4.46	271	5.33	.003	177	4.79	204	5.52	.257
	Middle third	990	14.97	858	16.89		625	16.91	590	15.96	
	Lower third	4402	66.56	3251	63.98		2398	64.86	2374	64.21	
	Other	927	14.02	701	13.80		497	13.44	529	14.31	
Grade	Grade I	273	4.13	452	8.90	<.001	210	5.68	192	5.19	.374
	Grade II	2632	39.79	2402	47.27		1680	45.44	1627	44.01	
	Grade III	3586	54.22	2153	42.37		1745	47.20	1817	49.15	
	Grade IV	123	1.86	74	1.46		62	1.68	61	1.65	
Histologic	Squamous cell carcinoma	1998	30.21	1684	33.14	<.001	1250	33.81	1303	35.24	.352
	Adenocarcinoma	3935	59.50	2996	58.96		2128	57.56	2067	55.91	
	Other	681	10.30	401	7.89		319	8.63	327	8.85	
T stage	T1	1135	17.16	2247	44.22	<.001	947	25.62	1031	27.89	<.001
	T2	765	11.57	724	14.25		531	14.36	587	15.88	
	T3	3522	53.25	1500	29.52		1724	46.63	1470	39.76	
	T4	1192	18.02	610	12.01		495	13.39	609	16.47	
M stage	M0	4420	66.83	4118	81.05	<.001	2647	71.60	2773	75.01	.001
	M1	2194	33.17	963	18.95		1050	28.40	924	24.99	
Surgery	NO	3972	60.05	2611	51.39	<.001	2171	58.72	2204	59.62	.449
	Yes	2642	39.95	2470	48.61		1526	41.28	1493	40.38	
Radiation	NO	1923	29.07	2253	44.34	<.001	1243	33.62	1082	29.27	<.001
	Yes	4691	70.93	2828	55.66		2454	66.38	2615	70.73	
Chemotherapy	NO	1326	20.05	2181	42.92	<.001	1024	27.70	915	24.75	.004
	Yes	5288	79.95	2900	57.08		2673	72.30	2782	75.25	
Tumor size	1–25	981	14.83	1574	30.98	<.001	728	19.69	667	18.04	.087
	26–69	3808	57.57	2650	52.16		2140	57.88	2228	60.27	
	70–560	1825	27.59	857	16.87		829	22.42	802	21.69	
Marital status	Unmarried	2496	37.74	2072	40.78	.001	1530	41.38	1491	40.33	.356
	Married	4118	62.26	3009	59.22		2167	58.62	2206	59.67	

We used the x-tile v3.6.1 (Yale University) to determine the optimal cutoffs for tumor size and age. PSM = propensity score matching.

### 3.2. Survival analysis

**Figure 1.**
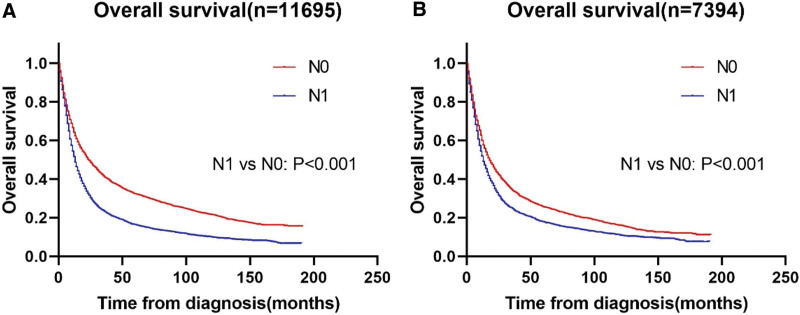
Kaplan–Meier curves of the pre- and post-PSM cohort. Kalpan–Meier curves of (A) the pre-PSM cohort, and (B) the post-PSM cohort. PSM = propensity score matching.

We matched a total of 3697 patients with LM with 3697 patients without LM by SPSS V26.0. The median survival in the post-PSM cohort was 14 months (interquartile range 6–45 months). Patient characteristics in the pre- and post-PSM cohorts are shown in Table 1. In the post-PSM cohort, 84.6% (n = 6262) of patients died during follow-up. The median OS was 13.0 (95% CI: 12.5–13.5) months and 22.0 (95% CI: 20.5–23.5) months for patients with EC with and without LM, respectively, in the pre-PSM cohort (Fig. 1A). The median OS was 13.0 (95% CI: 12.3–13.7) months and 17.0 (95% CI: 15.8–18.2) months for patients with EC with and without LM, respectively, in the post-PSM cohort (Fig. 1B).

### 3.3. Independent prognostic factors of LM in EC

We included patients with EC from 2005 to 2007, 2009 to 2011, and 2013 to 2015 in the training cohort (n = 5039) and patients diagnosed in 2004, 2008, and 2012 in the test cohort (n = 1575). All characteristics of the training and test sets are shown in Table 2. Using SPSS software, we included variables with *P* < .05 in univariate Cox regression into multivariate Cox regression and finally determined that variables including sex, M stage, surgery, histological type, race, grade, tumor size, T stage, radiotherapy, marital status, and chemotherapy were independent prognostic factors for LM of EC. More details are provided in Table 3.

**Table 2:** All characteristics of the training and test set.

		The training cohort		The validation cohort		*P*
		N	%	N	%	
		5039		1575		
Age	23–67	3213	63.76	1003	63.68	.558
	68–74	1011	20.06	302	19.17	
	75–85	815	16.17	270	17.14	
Race	White	4258	84.50	1348	85.59	.233
	Black	453	8.99	120	7.62	
	Other	328	6.51	107	6.79	
Gender	Female	859	17.05	259	16.44	.590
	Male	4180	82.95	1316	83.56	
Primary Site	Upper third	225	4.47	70	4.44	.741
	Middle third	762	15.12	228	14.48	
	Lower third	3358	66.64	1044	66.29	
	Other	694	13.77	233	14.79	
Grade	Grade I	211	4.19	62	3.94	.954
	Grade II	2010	39.89	622	39.49	
	Grade III	2724	54.06	862	54.73	
	Grade IV	94	1.87	29	1.84	
Histologic	Squamous cell carcinoma	1520	30.16	478	30.35	.023
	Adenocarcinoma	3028	60.09	907	57.59	
	Other	491	9.74	190	12.06	
T stage	T1	847	16.81	288	18.29	.063
	T2	577	11.45	188	11.94	
	T3	2729	54.16	793	50.35	
	T4	886	17.58	306	19.43	
M stage	M0	3389	67.26	1031	65.46	.188
	M1	1650	32.74	544	34.54	
Surgery	No	3046	60.45	926	58.79	.250
	Yes	1993	39.55	649	41.21	
Radiation	No	1414	28.06	509	32.32	.001
	Yes	3625	71.94	1066	67.68	
Chemotherapy	No	948	18.81	378	24.00	<.001
	Yes	4091	81.19	1197	76.00	
Tumor size	1–25	747	14.82	234	14.86	.955
	26–69	2906	57.67	902	57.27	
	70–560	1386	27.51	439	27.87	
Marital status	Unmarried	1932	38.34	564	35.81	.074
	Married	3107	61.66	1011	64.19	

We used the x-tile v3.6.1 (Yale University) to determine the optimal cutoffs for tumor size and age.

**Table 3:** Independent prognostic factors for LM of esophageal cancer.

		Overall survival			
		Univariate		Multivariate	
		HR (95%CI)	*P*	HR (95%CI)	*P*
Age	23–67	1		1	
	68–74	1.139 (1.056–1.229)	.001	1.110 (1.028–1.198)	.007
	75–85	1.511 (1.395–1.638)	<.001	1.225 (1.127–1.331)	<.001
Race	White	1		1	
	Black	1.444 (1.306–1.596)	<.001	1.148 (1.028–1.282)	.014
	Other	1.116 (0.989–1.260)	.075	1.035 (0.914–1.172)	.587
Gender	Female	1			
	Male	1.074	.078		
Primary Site	Upper third	1		1	
	Middle third	1.103 (0.941–1.292)	.226	1.183 (1.008–1.389)	.039
	Lower third	0.875 (0.757–1.011)	.070	1.096 (0.937–1.282)	.250
	Other	1.114 (0.949–1.308)	.185	1.172 (0.995–1.381)	.057
Grade	Well differentiated; Grade I	1		1	
	Moderately differentiated; Grade II	1.150 (0.983–1.347)	.082	1.231 (1.051–1.442)	.010
	Poorly differentiated; Grade III	1.375 (1.177–1.606)	<.001	1.428 (1.221–1.670)	<.001
	Undifferentiated; Grade IV	1.525 (1.175–1.978)	<.001	1.207 (0.926–1.573)	.164
Histologic	Squamous cell carcinoma	1		1	
	Adenocarcinoma	0.850 (0.796–0.908)	<.001	1.124 (1.034–1.222)	.006
	Other	1.069 (0.960–1.191)	.221	1.268 (1.125–1.429)	<.001
T stage	T1	1		1	
	T2	0.614 (0.547–0.689)	<.001	0.821 (0.729–0.924)	.001
	T3	0.762 (0.702–0.827)	<.001	1.032 (0.947–1.124)	.476
	T4	1.430 (1.296–1.578)	<.001	1.389 (1.256–1.537)	<.001
M stage	M0	1		1	
	M1	2.094 (1.967–2.230)	<.001	1.488 (1.385–1.598)	<.001
Surgery	No	1		1	
	Yes	0.436 (0.410–0.465)	<.001	0.509 (0.473–0.548)	<.001
Radiation	No	1		1	
	Yes	0.591 (0.554–0.630)	<.001	0.836 (0.766–0.901)	<.001
Chemotherapy	No	1		1	
	Yes	0.398 (0.370–0.428)	<.001	0.407 (0.374–0.443)	<.001
Tumor size	1–25	1		1	
	26–69	1.245 (1.139–1.361)	<.001	1.123 (1.025–1.231)	.013
	70–560	1.721 (1.562–1.897)	<.001	1.379 (1.246–1.527)	<.001
Marital status	Unmarried	1		1	
	Married	0.799 (0.753–0.849)	<.001	0.892 (0.838–0.949)	<.001

CI = confidence interval, HR = hazard ratio, LM = lymphatic metastasis.

### 3.4. Construction and verification of the nomogram

**Figure 2.**
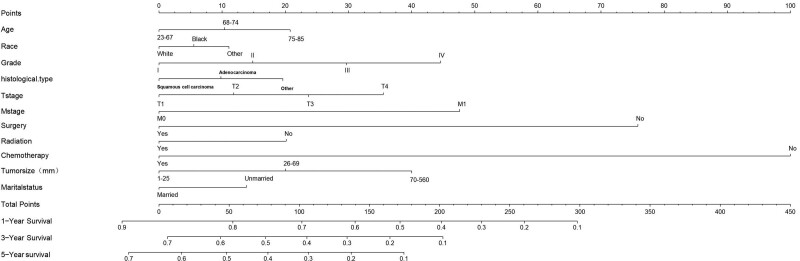
ROC curves of the nomogram. ROC curves of the nomogram to predict 1-, 3-, and 5-yr OS in the training cohort (A–C), and the internal validation cohort (D–F). FP = false positive rate, OS = overall survival, ROC = receiver operating characteristic, TP = true positive rate.

**Figure 3.**
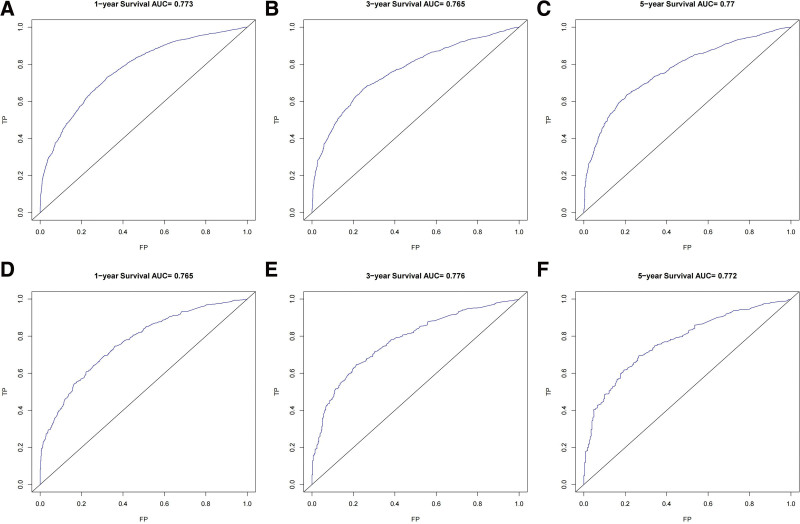
the calibration curves of the nomogram. The calibration curves of 1-, 3-, and 5-yr OS for esophageal cancer Lymphatic metastasis patients in the training cohort (A–C), and the internal validation cohort (D–F). The closer the dashed line and the blue solid line are, the more accurate the model is. OS = overall survival.

According to the results of multivariate Cox regression, a nomogram was built that could accurately predict the prognosis of EC after LM was constructed using R software v4.1.3. The C-index values are highly consistent, both 0.709 (95% CI: 0.701–0.717; 0.695–0.723), indicating that the model has excellent stability in the training set and test set. ROC analysis showed that the area under the curve values for 1-, 3-, and 5-year OS in the training cohort reached 0.773, 0.765, and 0.770, respectively (Fig. 2A–C). In the validation cohort, they were 0.765, 0.776, and 0.772, respectively (Fig. 2D–F). Calibration plots manifested good consistency between predicted survival and actual survival in both the training set (Fig. 3A–C) and the test set (Fig. 3D–F). The model accuracy in predicting OS is assessed by how close the solid blue line is to the dashed line.

## 4. Discussion

EC is a malignant tumor with poor prognosis and certain mortality in the chest. Symptoms such as esophageal obstruction appear late and usually reach the locally advanced stage or even the metastatic stage at the time of diagnosis.^[15]^ The prognosis for EC remains poor because it often spreads through its unique lymphatic system. Lymphatic status is an important observation indicator that affects patient prognosis in EC.^[16]^ Previous studies have found that approximately 52% of EC patients had LM.^[7]^ In our study, 56.5% (n = 6614) of patients had LM, and the median survival time with LM was remarkably lower than that without LM (*P* < .001). To exclude the influence of other variables, we performed 1:1 PSM. Despite some limitations, such as the inability to analyze and balance all variables, the confounding bias in observational studies can be reduced by PSM.^[17]^ In the post-PSM cohort, although the median survival time with LM was obviously lower, it was still remarkably higher than that of patients with LM (*P* < .001). Therefore, it is critical to determine the prognosis of EC patients with LM. Therefore, we conducted this study and constructed a nomogram. Furthermore, the calibration plots and ROC curves illustrated that the nomogram has considerable predictive power. This model will more easily guide clinical practice and enhance the comprehension of prognostic factors.

There have been several studies on the distant metastasis of EC. Shizhao Cheng et al^[18]^ found that bone metastasis, lung metastasis, and T stage were prognostic factors for brain metastasis of EC. Jin Zhang et al^[19]^ found that T stage, sex, marital status, brain metastases, and liver metastases were prognostic factors for bone metastases of EC. In our study, the prognostic factors for patients with LM from EC included sex, grade, race, surgery, T stage, chemotherapy, M stage, radiotherapy, tumor size, histological type, and marital status. Chemotherapy and surgery are the most important factors affecting the prognosis of patients with lymph-positive EC, and chemotherapy and surgery can significantly improve the survival of EC patients. Therefore, clinicians should try to consider chemotherapy and surgery for patients with lymph node metastasis.

**Figure 4.**
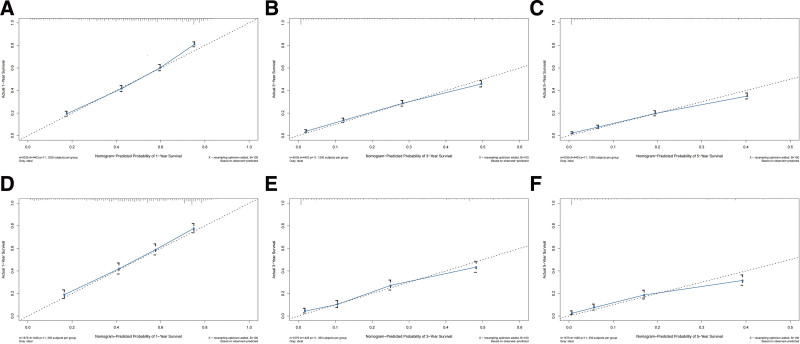
Nomogram for predicting the overall survival of patients with esophageal cancer Lymphatic metastasis. Nomogram for predicting the overall survival of patients with esophageal cancer Lymphatic metastasis. From this nomogram, the overall probability of survival at 1-, 3-, and 5-yr can be determined.

Compared with TNM staging, the nomogram is a simpler and more visual tool for estimating the risk based on patient characteristics and is widely used in oncology and medical prognosis.^[20]^ According to the results of multivariate Cox regression, we constructed a nomogram model for predicting the survival of patients with EC LM. It performs well on both the training and validation cohorts. As shown in Figure 4, EC patients with lymph node metastases who did not receive chemotherapy and surgery had significantly lower survival, so surgery and chemotherapy should be considered first for patients with resectable EC. In addition, age, T stage, race, M stage, grade, histological type, radiotherapy, tumor size, and marital status all affect the survival of EC patients with LM. Previous studies have found that marital status affects how long patients live. Some studies have found that marital status and distant metastasis affect patient survival.^[21,22]^

This study established a prognostic model that can be used to evaluate patients with EC after lymph node metastasis. The visualization feature of the nomogram is helpful for clinicians to make better judgments and target treatment. To verify the stability and effectiveness of the model, we divided all included data into a training set and a validation set according to the year of diagnosis, and the model showed great predictive capacity in both the training cohort and the test cohort.

This retrospective study inevitably has limitations. First, although the SEER database is the largest clinical database in the United States, it contains information on morbidity, mortality, and morbidity for approximately 30% of the U.S. population. However, some aspects of the information are incomplete, such as surgical methods, chemotherapy regimens, dose, radiation dose, and genetic information, which limits our further analysis. Second, there may be errors in the identification of patients with LM. LM can be confirmed by biopsy in only a minority of cases. When judging LM by CT or positron emission tomography-CT, it is difficult to avoid false positives and negatives. Third, the nomogram constructed in this study was not validated by an external validation cohort.

## 5. Conclusion

The survival time of EC patients with LM was remarkably lower than that of EC patients without LM. The nomogram model established in this study can precisely predict the survival of EC patients with LM.

## Acknowledgments

The authors thank the participants and their families in this study.

## Author contributions

**Conceptualization:** Feng Wang.

**Data curation:** Wenwen Yang, Yanjiang Yang.

**Formal analysis:** Li He.

**Funding acquisition:** Li He.

**Investigation:** Mengmeng Fan.

**Methodology:** Mengmeng Fan.

**Software:** Chang Chen.

**Supervision:** Mengmeng Fan, Biao Han.

**Validation:** Shuo Sun, Chang Chen.

**Visualization:** Shuo Sun, Biao Han.

**Writing – original draft:** Shuo Sun.

**Writing – review & editing:** Shuo Sun.
